# Association of 4p14 and 6q27 variation with Graves disease: a case–control study and a meta-analysis of available evidence

**DOI:** 10.1186/s12881-017-0406-7

**Published:** 2017-05-18

**Authors:** Fa-Mei Li, Lin Liu, Li-Nan Pang, Min Shen, Hong-Wen Lu, Xiao-Hong Zhang, Xun Chu, Zhen-ju Song

**Affiliations:** 1 Department of Clinical Medicine, Weifang Medical University, Shandong, People’s Republic of China; 2 Department of Endocrinology, Weifang People’s Hospital, Shandong, People’s Republic of China; 3 Xinhua Hospital, Shanghai Institute for Pediatric Research, Shanghai Jiao Tong University School of Medicine, 1665 Kongjiang Road, Shanghai, 200092 People’s Republic of China; 4 Department of Genetics, Shanghai-MOST Key Laboratory of Health and Disease Genomics, Chinese National Human Genome Center, Shanghai, People’s Republic of China; 5 Department of Emergency Medicine, Zhongshan Hospital, Fudan University, 180 Fenglin Road, Shanghai, 200032 People’s Republic of China

**Keywords:** Graves’ disease, Susceptibility, Single nucleotide polymorphisms, 4p14, 6q27

## Abstract

**Background:**

The etiology of the Graves’ disease (GD) is largely unknown. However, genetic factors are believed to play a major role. A recent genome-wide association study in a Han Chinese sample collection revealed two new Graves’ disease (GD) risk loci within chromosome band 4p14 and 6q27. In this study, we aimed to investigate these associations with Weifang Han Chinese population of Shandong province and perform a meta-analysis of associations with GD.

**Methods:**

A case–control study was conducted to investigate association of variation within 4p14 and 6q27 to GD susceptibility in Weifang Han Chinese population of Shandong province. SNP rs6832151 at chromosome 4p14 and SNP rs9355610 at chromosome 6q27 was selected for genotyping in 2,382 GD patients and 3,092 unrelated controls. SNP genotyping was performed using TaqMan Real-time PCR technique assays on ABI7900 platform. A meta-analysis was performed with the data obtained in the current sample-set and those available from prior studies.

**Results:**

Association analysis revealed both rs6832151 located in 4p14 (odds ratio (OR) = 1.27, *P*_*Allelic*_ = 1.48 × 10^−9^) and rs9355610 located in 6q27 (OR = 1.10, *P*_*Allelic*_ = 1.04 × 10^−2^) was associated with GD susceptibility. By model of inheritance analysis, we found the recessive model should be preferred (*P*_*Recessive*_ = 2.75 × 10^−11^) for rs6832151. The dominant model should be preferred (*P*_*Dominant*_ = 7.15 × 10^−3^) for rs9355610, whereas analysis of recessive model showed no significant association (*P*_*Recessive*_ = 0.13). Meta-analysis with the data of 10,781 cases and 16,304 controls obtained from present sample-set and those available from prior studies confirmed association of rs6832151 at 4p14 with GD susceptibility using a fixed model (OR = 1.27, 95% CI: 1.22 to 1.32; I^2^ = 0%). Meta-analysis with the data of 11,306 cases and 12,756 controls confirmed association of rs9355610 at 6q27 with GD susceptibility using a fixed model (OR = 1.18, 95% CI: 1.13 to 1.22; I^2^ = 41.2%).

**Conclusions:**

Our findings showed that chromosome 4p14 and 6q27 variants were associated with Graves’ disease in Weifang Han Chinese population of Shandong province.

## Background

Graves’ disease (GD) is a common organ-specific autoimmune disorder characterized by autoantibodies activating the thyrotropin receptor (TSHR) causing a hyperfunction of thyroid gland. Lymphocytic infiltration was found in the thyroid gland of patients with accompanying evidence of both humoral and cellular immune system activation. GD is clinically characterized by hyperthyroidism, diffuse goiter and the presence of thyrotropin receptor (TSHR) antibodies. Stimulatory TSHR auto-antibodies are directly responsible for the syndrome of hyperthyroidism in the development of GD.

Both genetic and environmental factors were thought to be involved in the pathogenesis of GD. Previous studies suggested that genetic background had a predominant impact on individual susceptibility to GD and contributed up to about 79% of total disease risk [1]. GD, as well as other common autoimmune disorders, emerges as a complex disease with multiple risk genes influencing the risk of morbidity. The well-established genetic risk genes predisposing to GD include human leukocyte antigen (*HLA*), cytotoxic T-lymphocyte associated antigen-4 (*CTLA-4*), *TSHR* and Fc receptor like 3 (*FcRL3*) [2–5].

A recent genome-wide association study (GWAS) in Chinese Han population firstly identified two novel GD susceptibility loci at chromosomal bands 4p14 (rs6832151) and 6q27 (rs9355610) [6]. Rs6832151 is located in an intergenic region at 4p14, which contained two annotated gene, namely *CHRNA9* and *RHOH*, and a newly cloned gene, namely *GDCG4p14.* Rs9355610 is located in a region of linkage disequilibrium (LD) at 6q27 containing three genes, namely *RNASET2*, *FGFR1OP* and *CCR6*. Rs9355610 lies 13-kb 5’ upstream *RNASET2*, which is the closest gene to rs9355610. The chromosome locations of these genes made them as the positional candidate genes for GD susceptibility.

The associations of variation at 4p14 and 6q27 were then investigated several following independent studies. Association of rs6832151 within 4p14 was replicated in Polish population, and rs9355610 within 6q27 was associated with GD susceptibility only following a recessive model in Polish population [7], whereas no significant association was found when comparing the difference of allele or genotype distribution between cases and controls. In study of Japanese population, rs9355610 within 6p27 was associated with Graves’ disease, whereas rs6832151 within 4p14 showed no significant associations [8]. Association with rs6832151 at 4p14 and rs9355610 at 6q27 were found with GD in several sample-sets of Chinese Han population from different regions [9–12]. These inconsistencies might be caused by population genetic heterogeneity or under powered sample size.

Because of possible genetic heterogeneity between Han Chinese of different regions, we investigated the associations of the above two SNPs (rs6832151 at 4p14 and rs9355610 at 6q27) with GD risk in Weifang Han population. To further clarify the inconclusive association between two SNPs and GD, we conducted a meta-analysis with available data sets from prior studies together with our current data.

## Methods

### Study population

The study population consisted of 2,382 Chinese Han GD patients and 3,092 unrelated, age and sex matched healthy controls. All patients with GD were recruited from Department of Endocrinology, Weifang People’s Hospital of Shandong province in China. The healthy controls were recruited from the Health Check-Up Center of the hospital, which were all of self-reported Chinese Han ethnicity from Weifang City of Shandong province. The study was approved by the ethics committee of Weifang People’s Hospital and written informed consent was taken from all participants.

Patients were diagnosed with GD based on the documented clinical symptoms and laboratory tests (increased thyroid hormone levels, decreased TSH levels and presence of TSH Receptor Antibodies (TRAb)) as described previously [13]. The thyroid function and autoantibody status were tested for all control subjects. Control subjects with subclinical autoimmune thyroid disease (AITD) and known family history of autoimmune disease were removed.

### DNA extraction and genotyping

Peripheral blood of 2 ml was collected from each participant. Genomic DNA was extracted from human peripheral blood cells using the FlexiGene DNA Kit 250 (Qiagen, Hilden, Germany) according to the manufactures’ guidelines. Two selected SNPs (rs6832151 and rs9355610) were genotyped using TaqMan assays on ABI7900 platform. TaqMan SNPs genotype assays were provided by Applied Biosystems (C__29224385_10 for rs6832151 and C__30614352_10 for rs9355610, respectively). SNP genotyping was performed on ABI7900 Sequence Detection System (Applied Biosystems, USA) according to the manufacturer’s instructions. The data completion rate of rs6832151 and rs9355610 was 99.6% and 99.4% respectively.

### Statistical analysis

Statistical analysis was performed using Plink. The genotype distribution of each SNP was tested for Hardy-Weinberg equilibrium in both case and control population. Both allele and genotype frequencies were assessed by *χ* ^2^-test between the cases and the controls. A two-tailed *P*-value < 0.05 was considered statistically significant. The risk allele of each SNP was revealed by odds ratios (ORs).

We assessed the power of the data using the CaTS [14]. Assuming disease prevalence of 1% and taking into account the expected risk allele frequency of rs6832151 (35%) and rs9355610 (49%) in the general population, the combined set of 2,382 GD cases and 3,902 controls provided a power of 99.8% and 74.5%, respectively, to support an association between GD and two SNPs, with an genotype relative risk of 1.2 and 1.1 respectively at the 5% significance level.

### Meta-analysis

To identify data to be included in the meta-analysis, a literature search was performed in PubMed (at http://www.pubmed.gov) up to April 2016. We searched for all publications relating to association studies and checked the reference lists of identified studies for additional studies. Association studies of chromosome 4p14 with GD were found by entering the search phrase: ‘GD’ or ‘Graves’ disease’ and ‘4p14’ and ‘rs6832151’ and ‘SNP’. Similarly, association studies of chromosome 6q27 with GD were found using the search phrase: ‘GD’ or ‘Graves’ disease’ and ‘6q27’ and ‘rs9355610’ and ‘SNP’. The analyzed data covered all English and Chinese publications from September 2011 to April 2016. Six studies including the present study investigated the association of rs6832151 at 4p14 with GD, and totally 10,781 cases and 16,304 controls were studied. Seven studies including the present study investigated the association of rs9355610 at 6q27 with GD, and totally 11,306 cases and 12,756 controls were studied.

We conducted meta-analysis using Review Manager software (version 4.2). The I^2^ statistic for inconsistency [15] and the *χ* ^2^ distributed Cochran Q-statistic [16] was used to assess heterogeneity across studies. I^2^ describes the proportion of variation that is unlikely due to chance and is considered significantly large for values > 50% [17]. Statistical significance of Q was accepted for *P*-values < 0.10. Fixed effects model using Mantel-Haenszel method was applied to pool the results since no heterogeneity was observed among studies (Q-test *P* > 0.100 and *I* ^2^ < 50%). All *P*-values are two-sided.

## Results

### Clinical characteristics of the samples

To investigate whether rs6832151and rs9355610 contributed to GD susceptibility, we recruited a case–control sample collection. Demographic information was shown in Table 1. The sex ratio was well matched between the cases and the controls. The ratio of female to male was 3.02 in GD patients and 2.96 in the healthy controls. The mean age of patients was 40.3 years and the mean age of healthy control subjects was 42.6 years.

**Table 1:** Demographic information for the samples

	Cases	Controls
Number of subjects	2382	3092
Female/Male	1790 / 592	2311/ 781
Average age at enrollment	40.3 ± 14.4	42.6 ± 11.7
Age range at enrollment	4-81	20-88

### Association analysis

In both of the patients and control samples, the distribution of genotype frequencies of the two SNPs conformed to Hardy-Weinberg equilibrium (*P* > 0.05). The genotypic frequencies of the two SNPs were showed in Table 2. Both rs6832151 and rs9355610 were associated with risk to GD.

**Table 2:** Case–control association analysis of the two SNPs

Chr.	SNP	Chr. Position	Genotype	Genotype distribution N(%)		Allelic		Genotypic	Dominant	Recessive
				Case	Control	OR (95% CI)	*P* value	*P* value	*P* value	*P* value
4	rs6832151	39998408	G/G	415 (17.5)	345 (11.2)	1.27(1.18– 1.38)	1.48 × 10^– 9^	5.65 × 10^−11^	1.78 × 10^−4^	2.75 × 10^−11^
			G/T	1093 (46.2)	1460 (47.5)					
			T/T	860 (36.3)	1271 (41.3)					
6	rs9355610	167303065	A/A	555 (23.4)	769 (25.2)	1.10(1.02 –1.19)	1.04 × 10^−2^	2.12 × 10^−2^	7.15 × 10^−3^	1.29 × 10^−1^
			A/G	1193 (50.3)	1578 (51.6)					
			G/G	626 (26.4)	709 (23.2)					

*SNP* single nucleotide polymorphism, *OR* odds ratio

The minor allele G of rs6832151 was associated with GD risk (*P*_*Allelic*_ = 1.48 × 10^−9^, OR = 1.27, 95% CI: 1.18-1.38). The frequency of rs6832151 G allele was 0.41in cases and 0.35 in controls. The frequencies of rs6832151 genotypes in GD patients (G/G, 17.5%; A/G, 46.2%, and A/A, 36.3%) differed significantly from those in the controls (G/G, 11.2%; A/G, 47.5% and A/A, 41.3%, respectively) (*P*_*Genotypic*_ = 5.65 × 10^−11^). Analysis of model of inheritance revealed the risk allele G of rs6832151 was associated with GD susceptibility following both the recessive model (*P*_*Recessive*_ = 2.75 × 10^−11^) and the dominant model (*P*_*Dominant*_ = 1.78 × 10^−4^). The recessive model should be preferred.

The major allele G of rs9355610 within 6q27 was associated with GD risk (*P*_*Allelic*_ = 1.04 × 10^−2^, OR = 1.10, 95% CI: 1.02-1.19). The frequency of rs9355610 risk allele G was 0.51 in cases and 0.49 in controls. The genotype frequencies of rs9355610 in GD cases (G/G, 26.4%; A/G, 50.3%, and A/A, 26.4%) differed significantly from those in the controls (G/G, 23.2%; A/G, 51.6% and A/A, 25.2%, respectively) (*P*_*Genotypic*_ = 2.12 × 10^−2^). Analysis of model of inheritance showed the G allele of rs9355610 had a dominant effect on GD in the current population (*P*_*Dominant*_ = 7.15 × 10^−3^), whereas analysis of recessive model showed no significant association (*P*_*Recessive*_ = 0.13).

### Meta-analysis

**Fig. 1:**
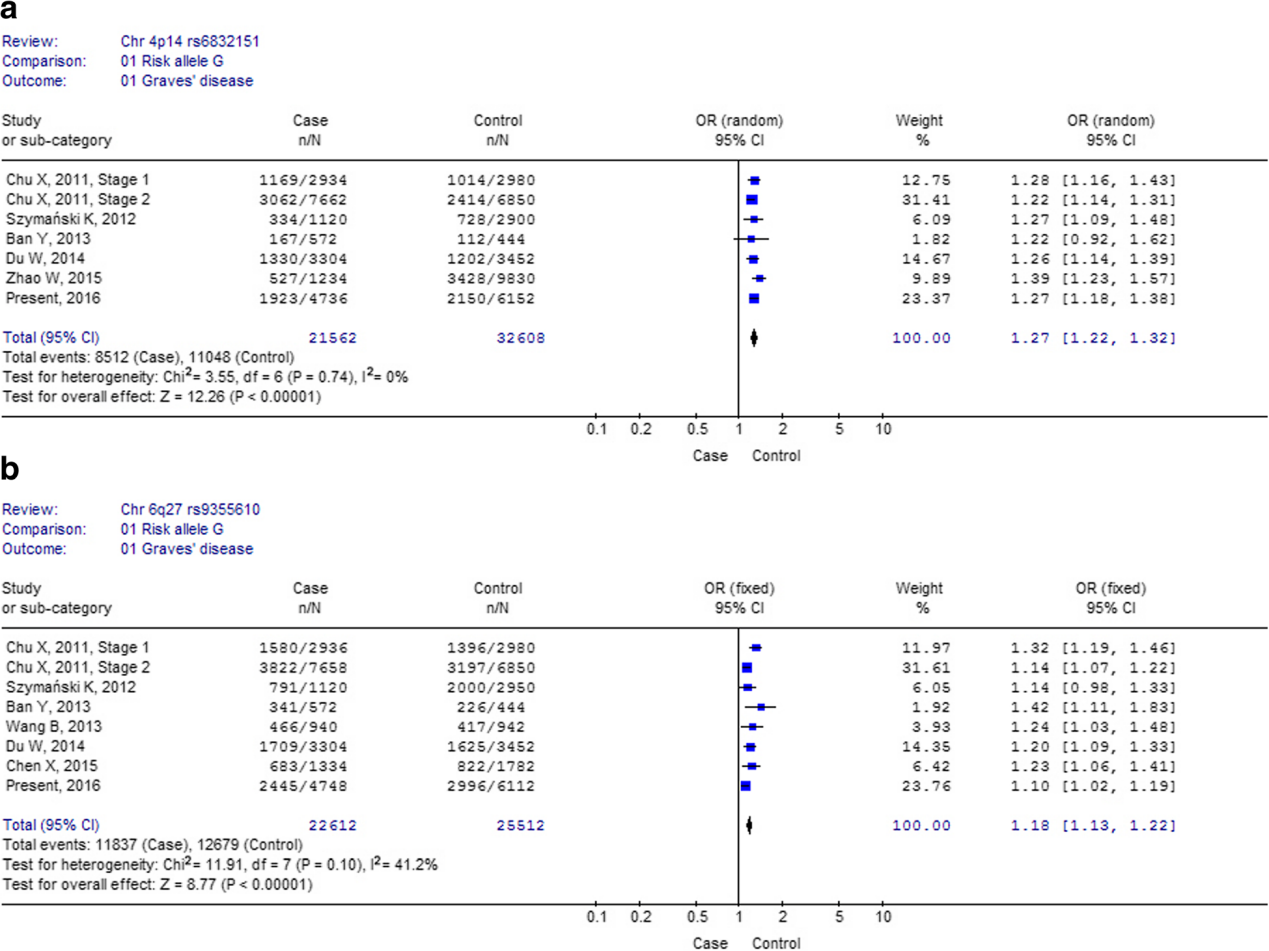
Forest plots of Meta-analysis for the association of rs6832151 and rs9355610 with GD susceptibility measured by allele frequency data. OR (*black squares*) and 95% CI (*bar*) are shown for each study. The pooled ORs and their 95% CIs are represented by the shaded diamonds. The symbol n indicates the total number of the risk alleles, and N indicates the total number of the risk alleles plus the protective alleles. **a** Meta-analysis of the association studies of rs6832151; **b** Meta-analysis of the association studies of rs9355610

Meta-analysis for rs6832151 and rs9355610 was performed combining the data from previous studies in Chinese, Polish Caucasian and Japanese populations, and the data of present study. Six studies including the present study investigated the association of rs6832151 at 4p14 with GD, and totally 10,781 cases and 16,304 controls were studied. Seven studies including the present study investigated the association of rs9355610 at 6q27 with GD, and totally 11,306 cases and 12,756 controls were studied. The allelic forest plots are shown in Fig. 1.

For meta-analysis of rs6832151 within 4p14, no heterogeneity was detected among the six studies (*P* = 0.74 for Q-test; I^2^ = 0%; Fig. 1a). The pooled OR was 1.27 (95% CI: 1.22 to 1.32) calculated by fixed effects approaches. The results confirmed the modest effect that rs6832151 within 4p14 played in GD susceptibility. The Japanese study showed no significant association for rs6832151 with GD risk (OR = 1.22; 95% CI: 0.92 to 1.62; Fig. 1a). However, the trend of the OR for the Japanese samples was similar to the Chinese and the Polish Caucasian samples. It might be that the sample size is too small to detect the genetic effect of rs6832151in the Japanese sample collection.

For meta-analysis of rs9355610 with 6q27, no significant heterogeneity presented among seven studies (*P* = 0.10 for Q-test; I^2^ = 41.2%; Fig. 1a). The pooled OR was 1.18 (95% CI: 1.13 to 1.22) calculated by fixed effects approaches. The Polish study showed no significant association for rs9355610 with GD risk individually (OR = 1.14; 95% CI: 0.99 to 1.33; Fig. 1b). However, the trend of the OR for the Polish samples was similar to the Chinese and the Japanese samples. It also might be that the sample size is not large enough to detect the moderate genetic effect of rs9355610 in the Polish sample collection.

## Discussion

In the current study, we replicated the associations of variation in chromosome band 4p14 and 6q27 with GD susceptibility in a North Han Chinese sample-set from Weifang City, which is a coastal city in Shandong province located in North China. Our meta-analysis including the data from previous studies together with the present data unequivocally replicated the association of GD susceptibility with rs9355610 and rs6832151. No significant heterogeneity was observed among the studies for both SNPs (Fig. 1). Of note, only one study in Caucasian population investigate these associations, further more studies in Caucasian and other population were needed to confirm these associations with GD susceptibility.

Combining seven data-sets from three countries across two continents, we were able to perform a meta-analysis for rs6832151 at 4p14 with association with GD that included 11,306 cases and 12,756 controls. The meta-analysis results confirmed the modest effect (OR = 1.27; 95% CI: 1.22 to 1.32) that the rs6832151 polymorphism at 4p14 played in GD susceptibility. Consistently positive associations were found in the six data-sets. However, no association was found Japanese population when comparing the allelic effects (OR = 1*.*22, 95%CI: 0.92–1.62, *P* = 0*.*17). The frequency of the risk allele G was 0.41 and 0.35 in the current Chinese cases and controls. The frequency of the risk allele G was 0.292 and 0.252 in the Japanese cohort with 286 cases and 222 controls. The trend of the ORs for each of the Chinese, Japanese and Polish cohorts was similar and there was no significant heterogeneity across the seven data-sets (Fig. 1a). It should be that the sample size is too small to detect the modest genetic effect of rs683215 with GD in the Japanese sample-set.

Rs6832151 is located within a 110-kb interval at 4p14. *CHRNA9*, *RHOH* and *GDCG4p14* are located near this region. Rs6832151 influenced the expression of mRNA of both *CHRNA9* and *GDCG4p14* [6]. However, these two neighboring genes harbor no SNPs in high LD with it. *CHRNA9* was found to be involved in various pathophysiologic processes, such as tumorigenesis, vestibulo-oculomotor interaction and chronic mechanical hyperalgesia [18–22]. Variation within *CHRNA9* region was associated with increased breast cancer risk and non-small cell lung cancer risk [23, 24]. Notably, the expression level of *CHRNA9* was relatively high in CD4^+^ and CD8^+^ T cells [6]. The newly cloned gene, *GDCG4p14*, had higher expression levels in CD4^+^ and CD8^+^ T cells. Therefore, both *CHRNA9* and *GDCG4p14* are positional and functional candidate genes for GD susceptibility. The expression of *RHOH* is limited to hematopoietic lineage cells [25]. RhoH is a key adapter protein that contributed to the regulation of both pre-T cell receptor (TCR) and TCR signaling during T cell development [26]. An excess amount of RhoH was able to initiate pre-TCR signaling in absence of pre-TCR complexes [27]. It could be hypothesized that *RHOH* might affect the T-cell-related immune response and thus play a role in pathogenesis of GD.

We also found rs9355610 in 6q27 was associated with GD risk in Weifang Han Chinese Han population. Our meta-analysis summarizes the evidence to date regarding the association between rs9355610 and GD, representing a pooled total of 11,306 cases and 12,756 controls from three countries across two continents. The results of meta-analysis indicated an association of rs9355610 with GD susceptibility (OR = 1.18; 95% CI: 1.13 to 1.22; Fig. 1b). Positive associations were found in the six data-sets. However, no association was found in Polish Caucasian population when comparing the allelic effects (OR = 1*.*14, 95% CI: 0.98–1.33, *P* = 0*.*082). The frequency of rs9355610 risk allele G was 0.51 and 0.49 in the current Chinese cases and controls. The frequency of the risk allele G was 0.71 and 0.68 in the Polish Caucasian cases and controls. The trend of the ORs for each of the Chinese, Japanese and Polish cohorts was similar and there is no significant heterogeneity across the seven data-sets (Fig. 1b). It should be that the sample size is not large enough to detect the genetic effect of rs683215 with GD in the Polish Caucasian sample-set of 560 GD patients and 1,475 controls.

LD pattern in Chinese population showed that rs9355610 was located in an LD block covering 5’ exons of *RNASET2* and the 5’ upstream region. The risk allele of rs9355610 was significantly with the diminished level of *RNASET2* expression in T cells from healthy subjects [6]. *RNASET2*, encoding ribonuclease T2, is the only RNase T2 family member in humans. Ribonucleases were suggested to have a broad range of biological functions including scavenging of nucleic acids, degradation of self-RNA, serving as cytotoxins and modulating host immune responses [28]. RNase T2 family members were found to be involved in the process of priming human dendritic cells for Th2 polarization of CD4^+^ T cells [29, 30]. The involvement of human *RNASET2* in immune response made it a potential GD susceptibility gene and further functional studies were needed to clarify whether and how it played a role in GD pathogenesis. It should be noted that the association of chromosome band 6q27 were also found with susceptibility of other autoimmune diseases such as Crohn’s disease, rheumatoid arthritis, type 1 diabetes mellitus and vitiligo [6], which suggested this locus should be a shared genetic region among common autoimmune diseases.

## Conclusions

We confirmed the association between GD susceptibility and rs6832151 at 4p14 as well as rs9355610 at 6q27. These associations indicated that variation in 4p14 and 6q27 might be involved in GD pathogenesis in Weifang Han Chinese population of Shandong province. Although our association study and meta-analysis replicated the associations of 4p14 and 6q27 variation with Graves’ disease with compelling evidence, future studies in Caucasian and other population were needed to further confirm these associations with GD susceptibility. Further molecular experiments should be put forth to clarify how these risk variants accounted for the susceptibility of GD.

## Abbreviations

GD: Graves’ disease
Kb: Kilobase pairs
MAF: Minor allele frequency
SNP: Single nucleotide polymorphism

## References

[1] Brix TH, Kyvik KO, Christensen K, Hegedus L (2001). Evidence for a major role of heredity in Graves’ disease: a population-based study of two Danish twin cohorts. J Clin Endocrinol Metab.

[2] Simmonds MJ, Howson JM, Heward JM, Cordell HJ, Foxall H, Carr-Smith J, Gibson SM, Walker N, Tomer Y, Franklyn JA (2005). Regression mapping of association between the human leukocyte antigen region and Graves disease. Am J Hum Genet.

[3] Ueda H, Howson JM, Esposito L, Heward J, Snook H, Chamberlain G, Rainbow DB, Hunter KM, Smith AN, Di Genova G (2003). Association of the T-cell regulatory gene CTLA4 with susceptibility to autoimmune disease. Nature.

[4] Dechairo BM, Zabaneh D, Collins J, Brand O, Dawson GJ, Green AP, Mackay I, Franklyn JA, Connell JM, Wass JA (2005). Association of the TSHR gene with Graves’ disease: the first disease specific locus. Eur J Hum Genet.

[5] Kochi Y, Yamada R, Suzuki A, Harley JB, Shirasawa S, Sawada T, Bae SC, Tokuhiro S, Chang X, Sekine A (2005). A functional variant in FCRL3, encoding Fc receptor-like 3, is associated with rheumatoid arthritis and several autoimmunities. Nat Genet.

[6] Chu X, Pan CM, Zhao SX, Liang J, Gao GQ, Zhang XM, Yuan GY, Li CG, Xue LQ, Shen M (2011). A genome-wide association study identifies two new risk loci for Graves’ disease. Nat Genet.

[7] Szymanski K, Bednarczuk T, Krajewski P, Ploski R (2012). The replication of the association of the rs6832151 within chromosomal band 4p14 with Graves’ disease in a Polish Caucasian population. Tissue Antigens.

[8] Ban Y, Tozaki T, Taniyama M (2013). The replication of the association of the rs9355610 within 6p27 with Graves’ disease. Autoimmunity.

[9] Wang BP, Han L, Tong JJ, Wang Y, Jia ZT, Sun MX, Wang HL (2013). Association of RNASET2 gene polymorphisms and haplotypes with Graves disease in Han Chinese population from coastal regions of Shandong. Zhonghua Yi Xue Yi Chuan Xue Za Zhi.

[10] Du W, Liang C, Che F, Liu X, Pan C, Zhao S, Dong Q, Li W, Wang Y, Pan Z (2014). Replication of association of nine susceptibility loci with Graves’ disease in the Chinese Han population. Int J Clin Exp Med.

[11] Zhao W, Sun W, Zhao S, Song H, Zhang X (2015). Association of the rs6832151 within chromosomal band 4p14 with Graves’ disease. CHIN J END MET.

[12] Chen XJ, Gong XH, Yan N, Meng S, Qin Q, Jiang YF, Zheng HY, Zhang JA (2015). RNASET2 tag SNP but not CCR6 polymorphisms is associated with autoimmune thyroid diseases in the Chinese Han population. BMC Med Genet.

[13] Skorka A, Bednarczuk T, Bar-Andziak E, Nauman J, Ploski R (2005). Lymphoid tyrosine phosphatase (PTPN22/LYP) variant and Graves’ disease in a Polish population: association and gene dose-dependent correlation with age of onset. Clin Endocrinol (Oxf).

[14] Skol AD, Scott LJ, Abecasis GR, Boehnke M (2006). Joint analysis is more efficient than replication-based analysis for two-stage genome-wide association studies. Nat Genet.

[15] Higgins JP, Thompson SG, Deeks JJ, Altman DG (2003). Measuring inconsistency in meta-analyses. BMJ.

[16] Lau J, Ioannidis JP, Schmid CH (1997). Quantitative synthesis in systematic reviews. Ann Intern Med.

[17] Higgins JP, Thompson SG (2002). Quantifying heterogeneity in a meta-analysis. Stat Med.

[18] Lee CH, Huang CS, Chen CS, Tu SH, Wang YJ, Chang YJ, Tam KW, Wei PL, Cheng TC, Chu JS (2010). Overexpression and activation of the alpha9-nicotinic receptor during tumorigenesis in human breast epithelial cells. J Natl Cancer Inst.

[19] Chen CS, Lee CH, Hsieh CD, Ho CT, Pan MH, Huang CS, Tu SH, Wang YJ, Chen LC, Chang YJ (2011). Nicotine-induced human breast cancer cell proliferation attenuated by garcinol through down-regulation of the nicotinic receptor and cyclin D3 proteins. Breast Cancer Res Treat.

[20] Shih YL, Liu HC, Chen CS, Hsu CH, Pan MH, Chang HW, Chang CH, Chen FC, Ho CT, Yang YY (2010). Combination treatment with luteolin and quercetin enhances antiproliferative effects in nicotine-treated MDA-MB-231 cells by down-regulating nicotinic acetylcholine receptors. J Agric Food Chem.

[21] Eron JN, Davidovics N, Della Santina CC (2015). Contribution of vestibular efferent system alpha-9 nicotinic receptors to vestibulo-oculomotor interaction and short-term vestibular compensation after unilateral labyrinthectomy in mice. Neurosci Lett.

[22] Mohammadi S (2014). Christie MJ: alpha9-nicotinic acetylcholine receptors contribute to the maintenance of chronic mechanical hyperalgesia, but not thermal or mechanical allodynia. Mol Pain.

[23] Hsieh YC, Lee CH, Tu SH, Wu CH, Hung CS, Hsieh MC, Chuang CW, Ho YS, Chiou HY (2014). CHRNA9 polymorphisms and smoking exposure synergize to increase the risk of breast cancer in Taiwan. Carcinogenesis.

[24] Wang Y, Zhang Y, Gu C, Bao W, Bao Y (2014). Neuronal acetylcholine receptor subunit alpha-9 (CHRNA9) polymorphisms are associated with NSCLC risk in a Chinese population. Med Oncol.

[25] Oda H, Tamehiro N, Patrick MS, Hayakawa K, Suzuki H (2013). Differential requirement for RhoH in development of TCRalphabeta CD8alphaalpha IELs and other types of T cells. Immunol Lett.

[26] Wang H, Zeng X, Fan Z, Lim B (2011). RhoH modulates pre-TCR and TCR signalling by regulating LCK. Cell Signal.

[27] Tamehiro N, Oda H, Shirai M, Suzuki H (2015). Overexpression of RhoH Permits to Bypass the Pre-TCR Checkpoint. PLoS One.

[28] Luhtala N, Parker R (2010). T2 Family ribonucleases: ancient enzymes with diverse roles. Trends Biochem Sci.

[29] Everts B, Perona-Wright G, Smits HH, Hokke CH, van der Ham AJ, Fitzsimmons CM, Doenhoff MJ, van der Bosch J, Mohrs K, Haas H (2009). Omega-1, a glycoprotein secreted by Schistosoma mansoni eggs, drives Th2 responses. J Exp Med.

[30] Steinfelder S, Andersen JF, Cannons JL, Feng CG, Joshi M, Dwyer D, Caspar P, Schwartzberg PL, Sher A, Jankovic D (2009). The major component in schistosome eggs responsible for conditioning dendritic cells for Th2 polarization is a T2 ribonuclease (omega-1). J Exp Med.

